# Comparison of the efficacy of static versus articular spacers in two-stage revision surgery for the treatment of infection following total knee arthroplasty: a meta-analysis

**DOI:** 10.1186/s13018-017-0644-6

**Published:** 2017-10-17

**Authors:** Hai Ding, Jian Yao, Wenju Chang, Fendou Liu

**Affiliations:** Department of Orthopedics, The First Affiliated Hospital of Bengbu Medical College, No. 287 Changhuai Road, Bengbu, Anhui 233004 People’s Republic of China

**Keywords:** Total knee arthroplasty, Periprosthetic infection, Two-stage revision arthroplasty, Antibiotic bone cement spacer, Meta-analysis

## Abstract

**Background:**

The aim of this study was to compare the outcomes of static versus articular spacers in two-stage reimplantation for the treatment of infected total knee arthroplasty (TKA).

**Methods:**

The literature regarding the articulating and static spacers for treating infected TKA were searched in PubMed, Embase, Cochrane Library, Chinese Periodical Full-Text Database of CNKI, and Wanfang database. Data were extracted according to the inclusion and exclusion criteria and analyzed by Review Manager 5.3.

**Results:**

Ten studies were included to this meta-analysis (nine retrospective studies, one prospective study) according to the principle of PICOS. There was no significant difference regarding the eradication rate (*P* = 0.28) and the American Knee Society knee score (KSS) pain score (*P* = 0.11) between the articulating and static spacers in the two-stage revision surgery. There was no significant difference regarding quadriceps femoroplasty and tibial tubercle osteotomy between the two groups (*P* = 0.50). The knee range of motion (ROM), Hospital for Special Surgery (HSS) score, and KSS function score in the articulating group were significantly higher than those in the static group (*P* < 0.00001).

**Conclusion:**

Articulating spacers can provide better ROM and knee function scores after revision surgery when compared to static spacer while not compromising the infection eradication rate, soft tissue contracture during exclusion period, and knee pain scores.

## Background

The prosthetic joint infection (PJI) is a devastating and complex complication after total knee arthroplasty (TKA). Although the incidence was only 1 to 2% [1–3], the number of the joint arthroplasties being performed is increasing. It can lead to serious consequences such as bone defect and necrosis; thus, the diagnosis and effective treatment of postoperative infection are very important. The two-stage revision surgery is the “gold standard” for patients with infection following TKA, especially for patients with chronic infection. The implantation of antibiotic-containing bone cement spacers can be used to eradicate infected microbes before prosthesis implantation [4, 5]. In addition, spacers maintain length of involved limbs and prevent muscle and soft tissue contracture via simple bone cement blocks inserting into the joint space [6–8]. Two types of antibiotic bone cement spacer are available, including articulating spacers and statics spacers. The articulating antibiotic-loaded cement spacer can be used to make femoral and tibial prosthesis via the artificial plastic method. This type of spacer is similar to the anatomical structure of the normal knee joint, and thus has a good match with the residual bone surface. Meanwhile, it can maintain joint space and joint activity, reduce soft tissue adhesion, atrophy and scar formation, and reduce the recurrence rate of infection [6, 9–11]. The static spacers can not only effectively deliver high concentration of antibiotic to control infection, but also maintain the joint space and limb length. However, the flexion and extension of the knee joint can result in soft tissue contracture around the joint and difficulty with reimplantation [1, 12]. The use of articular bone cement spacer is becoming increasingly widespread. However, some researchers reported higher risks of complications and the infection rate for the articular bone cement spacers compared to static spacers [13, 14]. Currently, there exists controversy regarding which antibiotic spacers are superior in the treatment of infection following total knee arthroplasty. The aim of the present meta-analysis was to compare the clinical outcomes of static spacers with mobile spacers for the treatment of infection following TKA. This paper was conducted in strict accordance with the PICOS principle of formulating the inclusion criteria, and the related literatures were collected and retrieved at home and abroad.

## Methods

### Search strategy

**Fig. 1:**
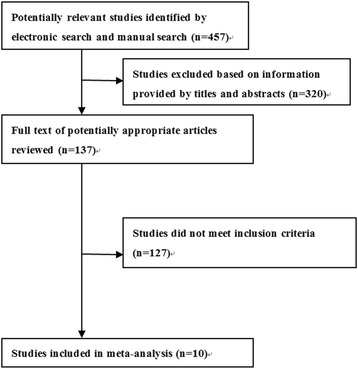
Flowchart of literature search

We searched the (“Total knee arthroplasty” OR “TKA” OR “joint replacement”) AND (“periprosthetic joint infection” OR “infection”) AND (“two-stage revision” OR “revision”) AND (“antibiotic bone cement spacer” OR “Spacers” OR “articulating Spacer” OR “static Spacer”) in PubMed, EMBASE and the Cochrane Library during January 1960 and October 2016. No regional and ethnic restriction was employed. All the subjects were humans. The detailed search process is shown in Fig. 1.

### Inclusion and exclusion criteria

Studies were considered to be eligible if they met the following criteria: (1) randomized controlled study, retrospective case-control study, or prospective cohort study of the two-stage revision of the first complete knee arthroplasty (which may include a small number of patients after knee revision surgery); (2) the study contains articulating group and static group; (3) the studies contains at least one of the following measured indicators: number of infection eradication, soft tissue contracture after the two-stage of surgery, recurrence of infection during the period of postoperative follow-up, range of motion after operation, postoperative follow-up joint function scores, and the complications (bone loss, mechanical complications, etc.).

Studies were excluded if they met the following criteria: (1) the total number of samples less than 20 cases of the study; (2) review literature, no control group literature, medical records reported literature; (3) the mean follow-up time less than 12 months; (4) studies from the same authors or repetitive reports; (5) poor quality observational studies; (6) non-English literature.

### Data extraction

Literature data were extracted by Professor Ding Hai independently, and another two assistants validated these literatures. Any disagreement between reviewers was resolved by Professor Ding Hai. The data extracted included the first author, publication time, the type of study, the sample size, the patient’s age, the average follow-up time, the type of antibiotic, the type of arthroplasty, the degree of preoperative joint activity, the degree of joint activity after revision at the final follow-up, number of recurrent infection at the final follow-up, knee score at the final follow-up, number of soft tissue angioplasty in the two stages of surgery, and postoperative complications (e.g., loss of bone loss and mechanical complications) at the final follow-up.

### Quality evaluation

Among the ten included studies, nine were retrospective case-control and one was a prospective cohort study. Newcastle-Ottawa Scale (NOS) scale was used to evaluate the literature quality [15]. NOS adopt the semi-quantitative principle of the star system. Nine stars represented the highest quality followed by high quality (5 to 9 stars), and low quality between zero and four stars.

### Statistical analysis

Review Manager 5.3 software was used for data analysis. The count data was expressed as the rates or the composition ratio. The odds ratio (OR) and 95% confidence interval (CI) were used to calculate the effect size. The measurement data were recorded by the mean and standard deviation (SD). The difference of the postoperative curative effect was based on the mean difference (MD) and the 95% CI. Then, we drew the forest map. *P* < 0.05 was considered to be statistically significant. Heterogeneity was assessed with the chi-square test value and *I* ^2^ value. Chi-square value of less than 0.1 and *I* ^2^ > 50% denoted a large heterogeneity among the included literatures, and the random effects model (RE) can be used to calculate the effect indicators. Conversely, the fixed effect model (FE) will apply. It should be noted that the heterogeneity of the source should be assessed if there is significant heterogeneity among the subjects.

## Results

### Study selection

Four hundred fifty-seven potentially eligible literatures were identified based on electronic databases search. After reviewing the titles and abstracts, 320 literatures did not meet the inclusion criteria. Subsequently, the full texts of the remaining 137 literatures were examined carefully. After excluding literatures with incomplete data, non-controls and those which are duplicated, a total of ten studies were finally included in this study [6–8, 16–22]. All the included literatures were deemed suitable for our inclusion criteria in this meta-analysis. The search process is shown in Fig. 1.

### Literature characteristics

The characteristics of the included studies were summarized in Table 1. There were 236 spacers in the articulating group and 256 in the static group. The number of samples ranged from 29 to 115, the mean postoperative follow-up period was more than 12 months. The antibiotic type of bone cement is mainly vancomycin or tobramycin, and then gentamicin or erythromycin. Articulating spacers can be divided into three types according to the production process: bone cement-bone cement type, metal-polyethylene type, and original pseudo-body type. Among the ten literatures, there was cement-bone cement type in six literatures, metal-polyethylene type in one literature, original pseudo-type in two literatures, and three coexisting type 1 literature. The NOS scores for these documents showed greater than 5 points, belonging to higher quality literature. The baseline information of the specific documents is shown in Table 1.

**Table 1:** Study characteristics

		Number of knees							
Study	Type of study	Articulating spacer group(A)	Static spacer group(S)	Age (A/S years)	M/F	Follow-up (months)	Type of antibiotic	Type of articulating positioner	Literature quality (NOS)
Brunnekreef J 2013	Retrospective	26	9	61/58	15/20	12	Gentamycin/erythromycin	Metal -polyethylene	8
Johnson AJ 2012	Retrospective	34	81	62/61	NA	27/66	Vancomycin/tobramycin	Three different types	7
Choi HR 2012	Retrospective	14 (10)	33 (31)	64	23/24	58	Vancomycin/ tobramycin	Original implant-bone cement	7
Chiang ER 2011	Prospective	23 (22)	22 (21)	71/72	22/23	41/40	Vancomycin	Bone cement-bone cement	7
Park SJ 2010	Retrospective	16	20	66.5/60.2	4/32	36/29	Vancomycin/erythromycin	Bone cement-bone cement	8
Freeman MG 2007	Retrospective	48	28	64.9/71.2	NA	Total 71.2 62.2/86.6	Vancomycin/tobramycin	Bone cement-bone cement	6
Hsu YC 2007	Retrospective	21	7	–	NA	58/101	Vancomycin/tobramycin or gentamicin	Bone cement-bone cement	8
Jämsen E 2006	Retrospective	24 (22)	10 (8)	68/70	11/23	32	NA	Original implant-bone cement	8
Emerson RH 2002	Retrospective	22	26	65.1/65.7	17/31	45.6/90	Vancomycin/tobramycin	Bone cement-bone cement	8
Fehring TK 2000	Retrospective	30 (15)^a^	25	NA	NA	27/36	Tobramycin	Bone cement-bone cement	7

Note: (1) The number in brackets in the sample size is the actual number of patients who completed the second revision and implantation of the new prosthesis (excluding the last document); (2) “NA” indicates that no information is available in the literature

^a^Fifteen is the number of patients who have actually completed follow-up

### Clinical outcomes

#### Infection eradication

**Fig. 2:**
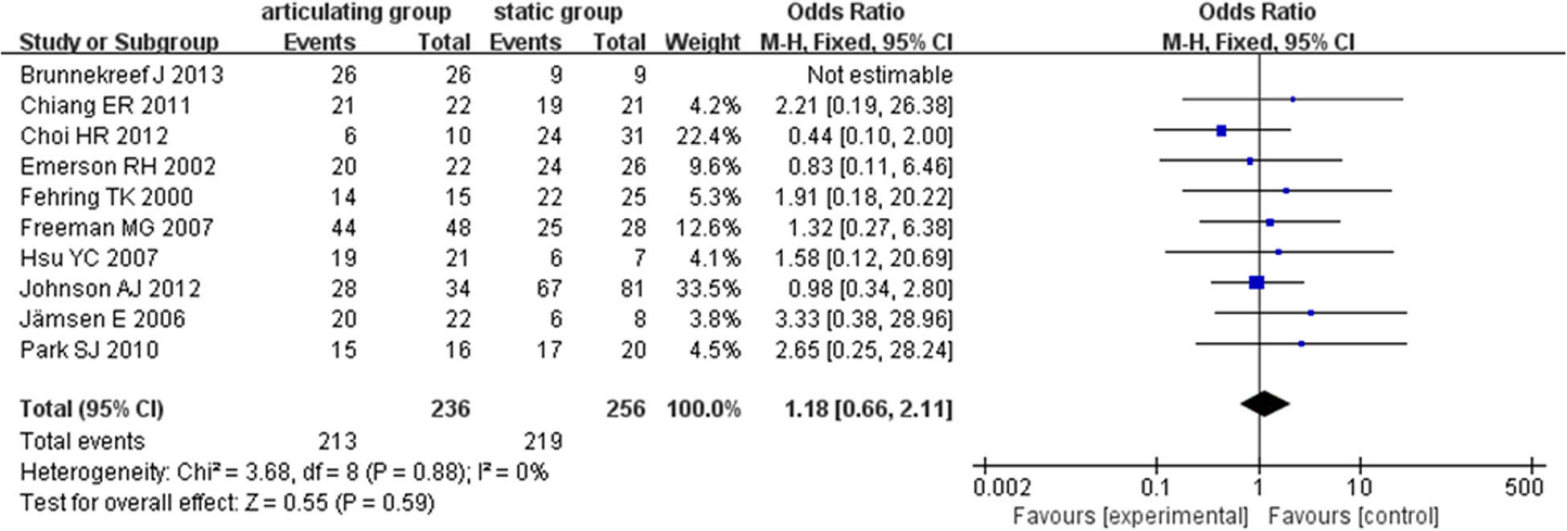
Forest plot diagram shows postoperative infection eradication between the two groups

Among ten articles were evaluated by the infection eradication [6–8, 16–22]. There was no significant heterogeneity among these included subjects (*χ* ^2^ = 3.68, *i* ^2^ = 0, *P* = 0.88). The fixed effect model was used for data analysis, and the results showed that there was no significant difference regarding the eradication rate between the articulating and statics spacers in the two-stage revision surgery of postoperative infection after TKA (OR = 1.18, 95% CI: 0.66~2.11, *P* = 0.59) Fig. 2).

### The release rate of soft tissue in two-stage revision (lengthening of the femoral quadriceps)

**Fig. 3:**
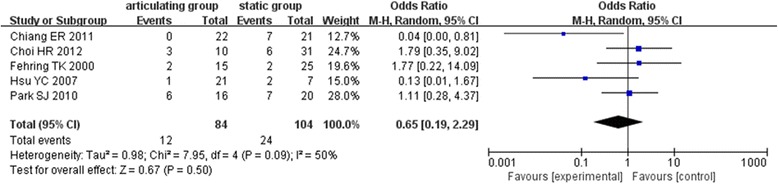
Forest plot diagram shows postoperative lengthening of the femoral quadriceps between the two groups

Among five literatures [7, 8, 18, 19, 21] were reported the release rate of soft tissue in two-stage revision (lengthening of the femoral quadriceps). There was moderate heterogeneity among the objects of study (*χ* ^2^ = 7.95, *I* ^2^ = 50%, *P* = 0.09) and using a random effects model. The results showed no significant difference regarding the release rate of soft tissue between articulating and static spacers in the two-stage revision of infection after TKA (OR = 0.65, 95% CI: 0.19~2.29, *P* = 0.50) (Fig. 3).

### The release rate of soft tissue in two-stage revision (tibial tubercle osteotomy)

**Fig. 4:**
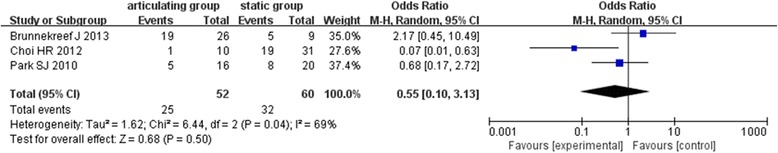
Forest plot diagram shows the tibial tubercle osteotomy between the two groups

Among three literatures were evaluated the release rate of soft tissue (tibial tubercle osteotomy) in two-stage revision of infection after TKA [8, 16, 18]. There was a big heterogeneity between the object of these studies (*χ* ^2^ = 6.44, *I* ^2^ = 69%, *P* = 0.04). Random effects model was used for analysis. The results showed no significant difference between articulating and static spacers in the two-stage revision of infection after TKA regarding soft tissue release rate (tibial tubercle osteotomy) (OR = 0.55, 95% CI 0.10 to 3.13, *P* = 0.50) (Fig. 4).

### Range of motion

**Fig. 5:**
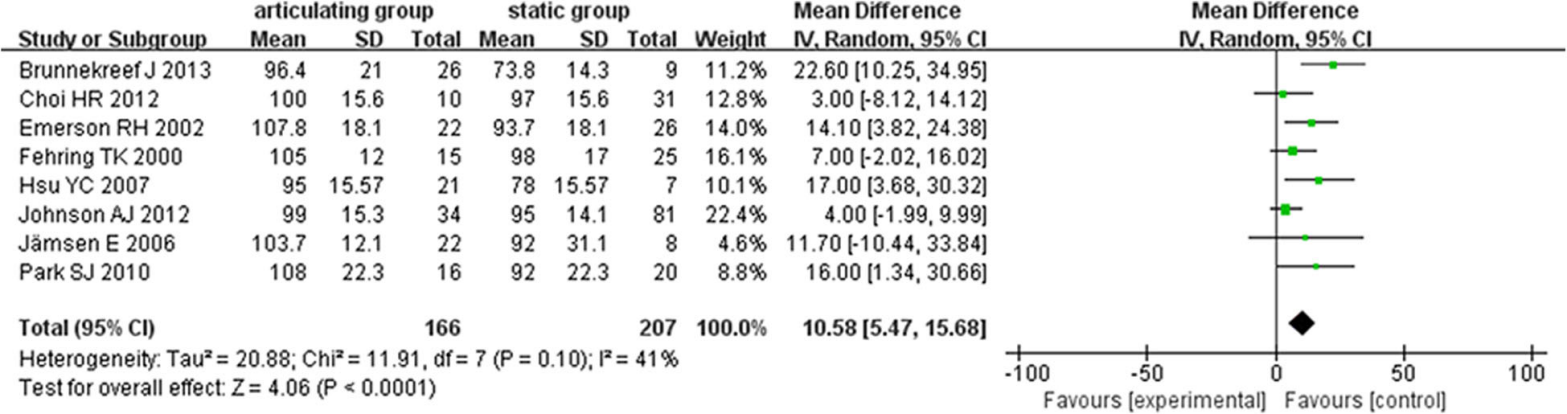
Forest plot diagram shows postoperative ROM between the two groups

Eight studies [6–8, 16–18, 21, 22] assessed the range of motion in this meta-analysis. There was a moderate heterogeneity among the subject in these studies (*χ* ^2^ = 16.73, *I* ^2^ = 52%, *P* = 0.03). Random effects model was used for analysis. The articulating group had greater postoperative ROM than the static group (MD = 12.19°, 95% CI 6.80~17.58, *P* < 0.00001) (Fig. 5).

### KSS (function) score

**Fig. 6:**
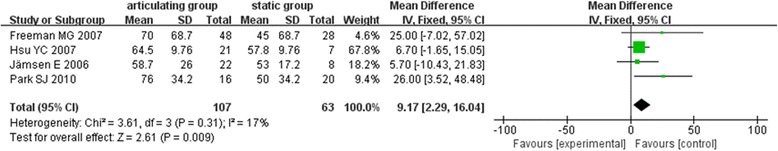
Forest plot diagram shows postoperative KSS (function) score between the two groups

Four studies assessed KSS (function) score [8, 20–22]. There was no significant heterogeneity between the object of these studies (*χ* ^2^ = 3.61, *I* ^2^ = 17%, *P* = 0.31). The fixed effect model was used for data analysis. There was a statistically significant difference between the articulating and static groups regarding KSS (function) score, and the articulating group had higher postoperative KSS (function) score than the static group (MD = 9.17, 95% CI 2.29~16.04, *P* = 0.009) (Fig. 6).

### KSS (pain) score

**Fig. 7:**
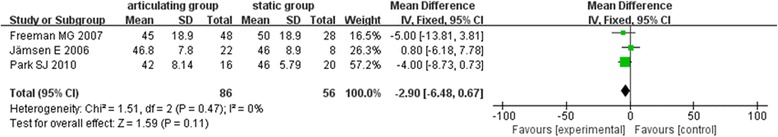
Forest plot diagram shows postoperative KSS (pain) score between the two groups

Only three studies reported KSS (pain) score [8, 20, 22]. There was no significant heterogeneity between the object of these studies (*χ* ^2^ = 1.51, *I* ^2^ = 0%, *P* = 0.47). The fixed effect model was used for data analysis. There was no statistically significant difference between the articulating and static groups regarding KSS (pain) score (MD = 2.90, 95% CI: − 6.48~0.67, *P* = 0.11) (Fig. 7).

### Hospital for Special Surgery (HSS) score

**Fig. 8:**
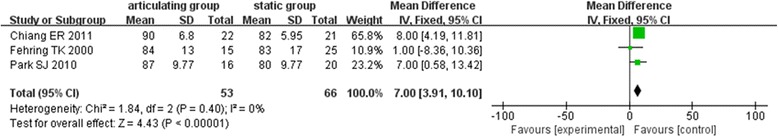
Forest plot diagram shows postoperative HSS score between the two groups

Three studies assessed HHS score [7, 8, 19] in the two-stage revision of infection after TKA. There was no significant heterogeneity between the object of these studies (*χ* ^2^ = 1.84, *I* ^2^ = 0%, *P* = 0.40). The fixed effect model was used for data analysis. There was a statistically significant difference between the articulating and static groups regarding HSS score, and the articulating group had higher postoperative HSS score than the static group (MD = 7.00, 95% CI: 3.91~10.10, *P* < 0.00001) (Fig. 8).

### Bone loss

There was less bone loss in the articulating group than in the static group [7, 8, 17, 21]. Fehring and Park reported that there was no bone loss in the articulating group [7, 8]. And Johnson and his colleague [17] demonstrated that more patients had severe bone loss in the static group (80%) than in the articulating group (53%). Femoral and tibial bone loss was 100% of the knees in the static group, while in the articulating group, only 28.6% of the knees with femoral bone loss, 47.6% of the knees with tibial bone loss [21].

### Publication bias

**Fig. 9:**
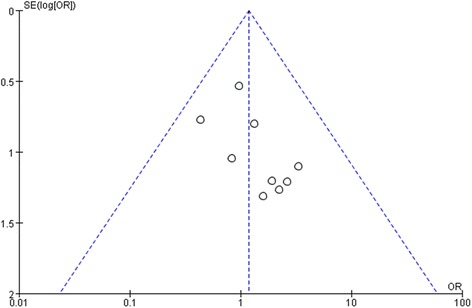
The graphical funnel plot

The graphical funnel plot may of included studies for outcome measurements appeared to be symmetrical (Fig. 9). The spots are evenly distributed on both sides of the inverted funnel, suggesting that there was no significant publication bias in the retrieved documents.

## Discussion

TKA has been regarded as an effective way to treat knee joint osteoarthritis, rheumatoid arthritis, and other advanced knee disorders since 1968. With the continuous improvement of surgical techniques and prosthetic design, TKA has become one of the most successful techniques in orthopedics. With the aging of the population, the demand for total knee replacement is still increasing. The increase in the amount of surgery is associated with increasing postoperative complications, of which PJI is one of the greatest complications. The current “gold standard” for the treatment of periprosthetic infection is still the two-stage revision surgery, which can fundamentally eliminate the infection and create healthy and vigorous tissue for the preparation of new prosthesis implantation [23]. During the two-stage revision, the doctor’s main goal is to prevent soft tissue contracture around the joint, which may lead to second operation exposure and refurbished prosthetic implant difficulties, and thus increased the surgical difficulties [24]. Therefore, it is crucial to maintain the stability of the knee joint and the balance of the soft tissue around the joint during the exclusion period of antibiotic bone cement. Moreover, there was a certain concentration of antibiotics in the knee joint to eradicate the infection [25, 26]. Currently, the antibiotic bone cement spacer can be divided into two categories, articulating type (hinge type) and static type (fixed type), and there was a significant controversy about whether articulating or static spacers can provide better outcomes. In this meta-analysis, we analyzed the difference of the two spacers regarding postoperative effects, including the infection eradication rate, the soft tissue release rate during the two-stage revision surgery, the ROM after the revision surgery, and function scores.

There was no significant difference between the two spacers regarding eradicate rate of infection in this study. The majority of the included articles reported the similar antibiotic use time, antibiotics for at least 6 weeks in vivo during the period of spacers [6–8, 16–18, 20–22]. There is no consistent standard for the best mix of methods for preparing high-dose antibiotic bone cement spacers. Most of the antibiotics associated with spacers mainly vancomycin (1~4 g of added 40 g bone cement) or gentamicin, tobramycin (2.4~4.8 g of added 40 g bone cement) [25]. Bone cement spacers should have a sufficiently high level of antibiotic to provide a relatively high concentration of local tissue release levels, while the dose of antibiotics should be low enough to prevent the mechanical properties of bone cement spacers weakened [27]. John F. Nettrour et al. [28] reported that there was no dose-dependent of antibiotic bone cement. Voleti et al. [29] compared the clinical effects between the articulating and static groups, 1526 patients were included in their systematic review (872 cases of articulating spacers and 654 cases of statics spacers). Their findings indicated no statistical significance difference regarding the infection eradication. The results of Voleti et al. study are consistent with Piver et al. [30] study. In contrast, Romano et al. [31] showed that the articulating spacers can achieve higher infection eradication than the static spacers (91.2 versus 87%). However, some authors suggested that the static spacer can provide greater release and space for the soft tissue around the joint infection or blood transfusion and can better eliminate the infection [32].

ROM and functional recovery are indicators of postoperative efficacy. In this meta-analysis, the results of ROM and KSS (function) score in articulating group significance were higher than in the static group. However, there was no significant difference regarding HSS and KSS (pain) score between the two groups. Dr. Javad Parvizi and Thorsten Gehrke concluded that there was an encouraging postoperative function in patients which were treated with articulating spacers in the two-stage revision of the knee surgery. In contrast, the increasing trend of ROM in statics spacers was higher than those patients with articulating spacers after follow-up for 2 years. Their results were consistent with some larger scale systematical reviews [29, 30, 33].

In this study, the soft tissue release rate during the two-stage revision surgery was used to assess whether there were differences regarding soft tissue contracture between the two different spacers. The results showed that there was no significant difference between either quadriceps or tibial tubercle osteotomy in two different spacers. However, Guild et al. [33] found that the patients with articulating spacers were less associated with the assistive technology than those with static spacers. The reason may be that knee activities during the exclusion period helps to maintain the length and flexibility of the extensor device and prevent the formation of scar tissue around the knee, quadriceps shortening, and joint capsule thickening and contracture [34]. The results of our study suggest no significant difference regarding the soft tissue release rate, which may be due to the relatively small number of participants and sample size. Randomized controlled studies with larger number of samples are still required.

Bone loss is also a common complication during the exclusion period. Guild et al. [33] reported that there was less bone loss in articulating group than the static group. A retrospective case-control study [8] reported that 15 (75%) patients had pre-existing tibial or femoral bone loss in the static group, 10 cases (65%) had pre-existing tibial bone loss, 13 (65%) patients had femoral bone loss, and 8 patients had tibial bone loss. However, there was no bone loss in the articulating group. Some researchers demonstrated that the static spacers did not restore the normal knee anatomical profile [35]. In the traditional “block” static spacers, the bone cement surface and the bone surface are in point-like contact; the uneven pressure distribution and the local high point pressure lead to a large amount of bone loss during the exclusion period. However, with the continuous updating of the static type of the design of the spacers, there has been “internal skeleton” type static spacer, and the stability of the knee joint will be further strengthened. Yoo et al. [36] reported that the four patients using an internal skeleton-type static spacer showed excellent clinical outcomes and no bone loss, and noted that this technique may be more suitable for patients with suspected bone loss after removal of the prosthesis. For these patients, the articulating spacers cannot provide sufficient stability. Most of the included literatures did not provide sufficient data for the bone loss in our research.

There are several limitations in this meta-analysis. (1) There were no RCTs in this article, and retrospective case-control studies inevitably lead to the recall bias and the confounding bias between subjects and will lead to objectivity influences. (2) The length of follow-up period in patients with static and articulating groups was different in some literatures. Each article had different follow-up time. Although the duration of follow-up was greater than 12 months, the deviation caused by different follow-up time may also affect the objectivity of the results. (3) All included studies did not specify any inclusion or exclusion criteria to determine which patients would receive the articulating spacers or the static spacers, resulting in considerable selection bias. (4) All the included patients in this study had larger age span. The differences in medical and health levels will lead to greater differences in postoperative efficacy. (5) During the data extraction of some continuous variable data, only the mean values were available in the literature; therefore, the standard deviation was obtained by averaging and the *P* value or 95% CI [37], which may lead to bias in the outcome and affect the objectivity. (6) Due to the limited number of literature, it was not possible to provide sufficient data for subgroup analysis, which may be one of the sources of heterogeneity.

## Conclusion

In this meta-analysis, we found no significant difference between articulating and static spacers in terms of infection eradication, the soft tissue contracture, and the knee pain scores. The patients with articulating spacers were able to achieve better ROM and limb function, but there was no significant difference in postoperative pain between the static and articulating spacers. Further randomized controlled studies on these two different spacers are still required to be carried out to provide more accurate and objective data for a comprehensive and accurate analysis.

## Abbreviations

CI: Confidence interval
FE: Fixed effect model
HSS: Hospital for special surgery knee score
KSS: American Knee Society knee score
MD: Mean difference
NOS: Newcastle-Ottawa Scale
OR: Odds ratio
PJI: Prosthetic joint infection
RE: Random effects
SD: Standard deviation
TKA: Total knee arthroplasty
